# A scoping review of emotion and non-cognitive measures of decision-making ability in older adults by the ARMCADA study

**DOI:** 10.3389/fpubh.2026.1718861

**Published:** 2026-02-04

**Authors:** Elizabeth M. Dworak, Sarah Pila, Miriam A. Novack, Zahra Hosseinian, Emily H. Ho, Berivan Ece, Tatiana Karpouzian-Rogers, Molly A. Mather, Y. Catherine Han, Patricia Bucko, David Cella, Richard C. Gershon, Sandra Weintraub

**Affiliations:** 1 Department of Medical Social Sciences, Northwestern University Feinberg School of Medicine, Chicago, IL, United States; 2 Department of Psychiatry and Behavioral Sciences, Northwestern University Feinberg School of Medicine, Chicago, IL, United States; 3 Mesulam Institute for Cognitive Neurology and Alzheimer’s Disease, Northwestern University Feinberg School of Medicine, Chicago, IL, United States

**Keywords:** aging, decision-making, emotion, risk-taking, scoping review

## Abstract

**Introduction:**

Decision-making is a complex form of cognitive function that declines with age and is highly susceptible to impairment from dementia due to Alzheimer’s and related neurogenerative diseases. Emotions and other non-cognitive assets are also believed to influence decision-making ability. The Advancing Reliable Measurement in Cognitive Aging and Decision-making Ability (ARMCADA) research initiative seeks to understand how measures of emotion and non-cognitive influences of decision-making (ENC DM) have been used in research and clinical settings. The scoping review examined the recent literature on decision-making measures involving emotion and non-cognitive domains in aging samples.

**Methods:**

Using a well-established scoping review methodology framework we conducted a systematic search across six databases—Embase (Elsevier), MEDLINE (Ovid), PsycINFO (EbscoHost), Cochrane Library, Web of Science (Clarivate), and Scopus (Elsevier)—to identify studies published between January 2018 and November 2023 that met our predefined eligibility criteria. In line with recommended best practices, sample selection was carried out in two-stages: First, two reviewers independently screened titles and abstracts for relevance; second, full-text articles were reviewed, and data were extracted from those that met the inclusion criteria.

**Results:**

The final dataset included 232 articles and, among them, 143 unique emotion and non-cognitive decision-making measures. Twenty-eight percent of manuscripts used measures with a clinical sample, 26% used measures with a normative adult sample, and 47% used measures with both clinical and non-clinical samples. The five most frequent measures used were: Iowa Gambling Task, Balloon Analogue Risk Task, Delay Discounting Task, Decisional Conflict Scale, and Cambridge Gambling Task.

**Discussion:**

Results of the most common decision-making measures reflect a preference for assessing risk-taking and impulsivity in the sphere of non-cognitive and emotion function. Such trends were also found in clinical samples of older adults with neurodegenerative diseases. These findings inform the larger ARMCADA project in the development of reliable and validated measurement of decision-making among older adults. It will also support researchers studying older adults’ decision-making skills and their vulnerability to diseases that cause cognitive impairment and dementia, with an understanding of the current assessments used to track ENC DM.

**Systematic review registration:**

https://bmjopen.bmj.com/content/14/12/e084178

## 1 Introduction

Assessment of cognitive function is complex. Cognition is a multifaceted construct, the components of which change across the lifespan due to both natural and pathological factors. Cognitive performance also often has a dynamic relationship with affect, personality, motivation, context, and other domains of functional status (e.g., fatigue, vision, hearing, motor)—constructs that are increasingly recognized as critical for characterizing at-risk states for cognitive decline and incident dementia.

Decision-making ability, a cognitive process that involves weighing possibilities and selecting a course of action, is especially susceptible to the direct and indirect influence of emotions and other non-cognitive influences (1, 2). This vulnerability is because decisions are made in diverse contexts, which may elicit distinct affective arousal and valence for individuals. Likewise, how individuals perceive different environments is primed by individual differences (3). Proper assessment of decision-making in the context of emotion and non-cognitive domains is essential for researching the impact of cognitive decline experienced by many adults as they age. The current paper aims to review the literature on available measures of emotion and non-cognitive influences on decision-making (ENC DM) ability to better understand the scope and range of measures used in aging samples.

Emotions, understood as “a collection of psychological states that include subjective experience, expressive behavior, and peripheral physiological responses.” (4), play a critical role in decision-making. Emotions and affect heuristics (mental shortcuts shaped by current emotion) influence decision-making by affecting risk perception, judgment, and choice preferences (1–3, 5, 6). For instance, emotions can bias decisions, leading to either overly cautious or excessively risky behavior depending on their valence. Positive emotions, such as happiness, tend to make people more risk-seeking, while negative emotions, like sadness or fear, can result in more risk-averse behavior (7).

Mood also affects temporal discounting, the tendency to prioritize immediate rewards over delayed ones. Individuals in a positive emotional state may discount future rewards less, whereas negative emotions can heighten the preference for immediate gains (8, 9). Emotion regulation, like emotions and affect themselves, also plays a critical role in decision-making. It moderates the relationship between emotional states and decision outcomes (10), and functions as a form of decision-making through the choices an individual makes in responding to emotional experiences (11). In the presence of reward cues, effective regulation strategies can reduce impulsive or risk-seeking behavior (12) and promote goal-directed choices, particularly when emotional arousal might otherwise lead to maladaptive decisions (13).

Beyond emotion, other non-cognitive factors shape how individuals interpret situations, weigh options, and ultimately behave. Stable traits, such as conscientiousness, neuroticism, impulsivity, and locus of control, affect dynamic behaviors like risk-taking, planning, and self-regulation. For example, individuals high in conscientiousness and low in neuroticism are more likely to delay gratification in favor of larger future rewards, while those with the opposite traits may prefer immediate outcomes (14, 98).

Individual differences in goal-directed behavior affect how people handle delayed gratification or weigh smaller immediate rewards against larger future benefits with some uncertainty (3). Factors such as tolerance for ambiguity and susceptibility to cognitive biases also influence how individuals process uncertainty (15, 16). Those more tolerant of ambiguity, for instance, may be more willing to pursue riskier options if the perceived benefit is greater (17, 18).

Social and interpersonal factors, including trust in others, peer influence, social norms, and cultural background, further shape decision-making by shaping values and creating social expectations (19). Situational influences such as time pressure, framing effects, environmental cues, and resource availability can also alter decisions without changing underlying preferences. While these factors provide a rich framework for understanding non-cognitive influences, the list is not exhaustive; decision-making is a complex and dynamic process shaped by a wide array of interacting influences.

Contemporary models of decision-making highlight the centrality of emotional and non-cognitive factors. The Emotion-Imbued Choice model (1) posits that emotional states shape perceived utility and decision strategies by altering outcome salience. Such emotions may distort an individual’s probability estimates, elevate or suppress one’s sensitivity to losses, bias temporal preferences, or change goals. Other integrative frameworks emphasize how non-cognitive influences, including personality traits and motivational goals, interact with core cognitive mechanisms to shape decision outcomes. Collins and Shenhav’s (20) model of learning and decision-making describes how reinforcement learning interacts with higher-order executive processes (e.g., planning and working memory) modulated by affective and emotional inputs. Emotional and social factors influencing attentional focus, learning rates, and strategy selection, thereby embedding decision-making within a broader network of affective and motivational inputs. This dynamic system allows for flexible, context-sensitive behavior, particularly under conditions of uncertainty.

Aging introduces further complexity. Löckenhoff’s (21) conceptual framework suggests older adults adapt to declining fluid cognitive abilities by relying more on heuristic and emotionally guided strategies, supported by preserved crystallized intelligence and enhanced emotion regulation. Motivational priorities also shift with age, often emphasizing emotional satisfaction and certainty. These adaptations may represent compensatory mechanisms that help older adults maintain decision competence in the face of cognitive change. Other contemporary models, such as the Affect-Integration-Motivation framework (22), suggest that structural and functional changes in key brain regions during aging alter risk sensitivity, reward valuation, and loss aversion in older adults. Rather than a uniform decline, decision-making in later life reflects rebalanced neural dynamics that may increase variability while also supporting strengths in future-oriented valuation and emotion-driven judgment. These frameworks collectively illustrate that decision-making is not simply a function of cognitive ability but emerges from a rich interplay between cognition, emotion, non-cognitive factors, and neural changes.

Understanding constructs related to decision-making ability and changes over time is crucial for studying decline caused by associated Alzheimer’s disease and related diseases that cause dementia (aka ADRD). Dysfunction or other rapid changes in mood or personality are symptoms that are significant indicators of dementia (23–25). It is estimated that nearly 61–97% of individuals with a dementia diagnosis experience these symptoms (26–28), although their specific manifestation can vary depending on the type of dementia, the severity of the illness, and its progression. Emotional symptoms are particularly common among individuals with clinical diagnoses of dementia due to Alzheimer’s disease, frontotemporal dementia, and vascular dementia.

In addition to emotional and behavioral changes, difficulties with decision-making can also signal cognitive decline. Decision-making difficulties can be one of the early indicators of cognitive decline, sometimes emerging before other symptoms become evident (29–31). Cognitive impairments also often disrupt emotional processing and alter personality traits, leading to decision-making that relies more on immediate emotions than on rational analysis. For example, cognitive decline has been associated with increased risk-taking behaviors, such as unwarranted trust in strangers (32) and a greater willingness to pursue risky treatments (33). Cognitive impairment has also been linked to changes in temporal discounting (34), such that individuals with cognitive decline may exhibit an increased preference for immediate rewards, even when delayed options offer greater long-term benefits. Likewise, diminished cognitive function is closely associated with elevated levels of impulsivity (35). These shifts in decision-making patterns can serve as an early marker for cognitive impairments, highlighting the potential for decision-making capacity to aid in the early detection and management of dementia and related conditions. However, even older adults without identified cognitive dysfunction can exhibit difficulties with decision-making ability (36, 37), emphasizing the need for sensitive assessment approaches that can differentiate between changes that may be normal for the stage of life from those that may indicate more pernicious cognitive decline. By integrating emotions, personality, social context, and other non-cognitive factors into decision-making research, we stand to gain a more nuanced understanding of these factors’ influence on cognitive health. This approach not only provides insights into the complexities of decision-making but also helps identify early signs of age-related cognitive decline, potentially allowing for earlier intervention and improved management of dementia and related conditions.

This study is embedded within the Advancing Reliable Measurement in Cognitive Aging and Decision-making Ability (ARMCADA) research initiative, a project aimed at developing efficient, psychometrically robust tools for assessing decision-making abilities in midlife and older adulthood. ARMCADA’s overarching goal is to create a brief (10- and 30-min) multidomain decision-making battery that can be used across diverse settings, including clinical, research, and community environments, to detect subtle changes in cognition and decision-making that may precede abnormal cognitive decline. By enabling earlier identification of emerging decision-making difficulties, the ARMCADA battery is intended to support early intervention and timely planning for individuals who may be on a trajectory to developing impaired cognition.

To inform the development of this battery, ARMCADA completed a comprehensive multi-domain scoping review (38) designed to document and evaluate existing decision-making (DM) measures used with adults aged 45 and older across a wide range of populations. While the larger scoping review examined tools spanning multiple target domains of decision-making [healthcare, financial, functional outcomes, end of life, and emotion (non-cognitive)] that are critical for understanding real-world decision behavior, the present study focuses specifically on the findings related to ENC assessments used in decision-making research. Unlike prior reviews that primarily targeted adults aged 50–60 years and older (39–42), our inclusion of adults aged 45 and older provides a broader perspective on ENC DM changes beginning in midlife. We also highlight measures used across both healthy and clinical populations to map current practices, identify gaps, and suggest refinements for decision-making capacity assessments. Such a demonstration will enable researchers and clinicians to select more appropriate and reliable measures, ultimately enhancing the accuracy of capacity assessments and supporting better clinical outcomes for individuals with cognitive impairments. By identifying measures that are sensitive, practical, and psychometrically robust within the ENC DM domain specifically, this review contributes directly to ARMCADA’s measure-selection process. Findings will help determine which existing measures, components, or administration methods are best suited for inclusion in the final ARMCADA battery, ensuring that the resulting tool captures the full spectrum of factors influencing everyday decision-making in aging populations.

## 2 Methods

### 2.1 Search criteria and eligibility

Our methodology followed Arksey and O'Malley's (43) scoping review framework. We searched six main databases: Embase (Elsevier), MEDLINE (Ovid), PsycINFO (EbscoHost), Cochrane Library (Wiley), Web of Science (Clarivate), and Scopus (Elsevier). The search criteria were iteratively designed to capture both broad and targeted domains of decision-making ability. To ensure comprehensive exploration of the topic, considering multiple aspects of decision-making, four teams of scientists independently proposed search terms based on their area of expertise. The proposed terms were then combined, reviewed, and refined by a larger research team. After this proposal phase, the terms selected were carefully reviewed by a panel of experts in decision-making, ensuring their relevance and accuracy. Lastly, feedback from our steering committee further refined and validated the criteria. This collaborative process strengthened the robustness and precision of the search parameters.

As part of the larger ARMCADA project, our overarching goal was to identify measures of decision-making skills in adults spanning ENC DM, functional outcomes (44), end of life (45), healthcare (46), financial (47), and general DM abilities. A list of the search terms specific for the ENC DM domain can be found in Table 1, however, identified articles used in this review came from a larger set of search terms provided in Ho et al. (38) and included terms potentially less relevant to ENC DM, to avoid missing indirect measures. Given the rich amount of data and differences between domain findings, we chose to discuss results in separate manuscripts dedicated to each specific domain. This manuscript describes findings related to ENC DM abilities. The review methodology and results are reported in accordance to the PRISMA Extension for Scoping Reviews [PRISMA-ScR; (48)].

**Table 1:** Emotion and non-cognitive domain specific search terms.

Search term
affect
affection
affective
affective valence
agitation
alienation
ambivalence
anger
anxiety
apathy
aversion
belonging
bereavement
boredom
catastrophizing
close relationships
compassion
conditioned emotional responses
contempt
contentment
couples
depression
desire
disappointment
disgust
dissatisfaction
distress
doubt
embarrassment
emotion
emotion*
emotional attachment
emotional content
emotional disturbances
emotional exhaustion
emotional health
emotional processing
emotional regulation
emotional responses
emotional states
emotional style
emotional support
emotional trauma
emotional wellbeing
emotions
empathy
enthusiasm
euphoria
euthymia
expressed emotion
family relations
fear
forgiveness
friendship
frustration
gratitude
greed
grief
guilt
happiness
helplessness
homesickness
hope
hopelessness
human relation
interpersonal relations
interpersonal relationships
jealousy
kinship
loneliness
love
mania
marital relations
mental confusion
mentor
morale
negative emotions
non cognitive
noncognitive
optimism
panic
partners
passion
peers
pessimism
pleasure
positive emotions
pride
prosocial behavior
prosocial behavior
psycholog*
psychological aspect
psychological engagement
regret
relationship quality
relationship termination
restlessness
role models
sadness
shame
significant others
social adjustment
social aspect
social behavior
social cognition
social interaction
social responsibility
social responsibility
social skills
social value
social values
solidarity
suffering
suspicion
sympathy

A * indicated a wildcard portion of a search. A full list of search terms used by this project can be found in the supplementary material of Ho et al. (38).

### 2.2 Protocol and registration

This work was registered under open science principles and full methods are described in the protocol by Ho et al. (38).

### 2.3 Screening

The articles were screened in Covidence (49) using predefined inclusion and exclusion criteria (Table 2). Study inclusion criteria were peer reviewed publications from January 2018 to December 2023 with samples aged 45 or over and mentioned at least one assessment of target domains [healthcare, financial, functional outcomes, end of life, and emotion (non-cognitive)]. We restricted our search to 2018 and 2023 to focus on contemporary measures of DM at the time of our review. Manuscripts in any language or country were eligible but excluded single-subject research/case studies or focus groups, narrative reviews, conference proceedings, book chapters, dissertations, commentaries, pre-prints, and other non-research publications.

**Table 2:** Overall inclusion and exclusion criteria for articles reviewed by the ARMCADA project.

Criteria domain	Inclusion criteria	Exclusion criteria
Population	Adults over age 45 The assessment was conducted with at least one group of individuals over 45 years Age range includes participants aged 45 and over	Adults ≤ 45 years old
Study characteristics	The study mentions at least one assessment of one or more of the target domains The domain of interest is an outcome assessed by the study.	Single-subject research/case studies Focus group Review articles Narrative reviews Gray literature Conference Proceedings Books and/or book chapters Commentaries Preprints Other non-research publications
Other	Language: All languages Location: All geographical locations	Articles that only measure shared decision-making Articles that only measure decision aids

**Figure 1:**
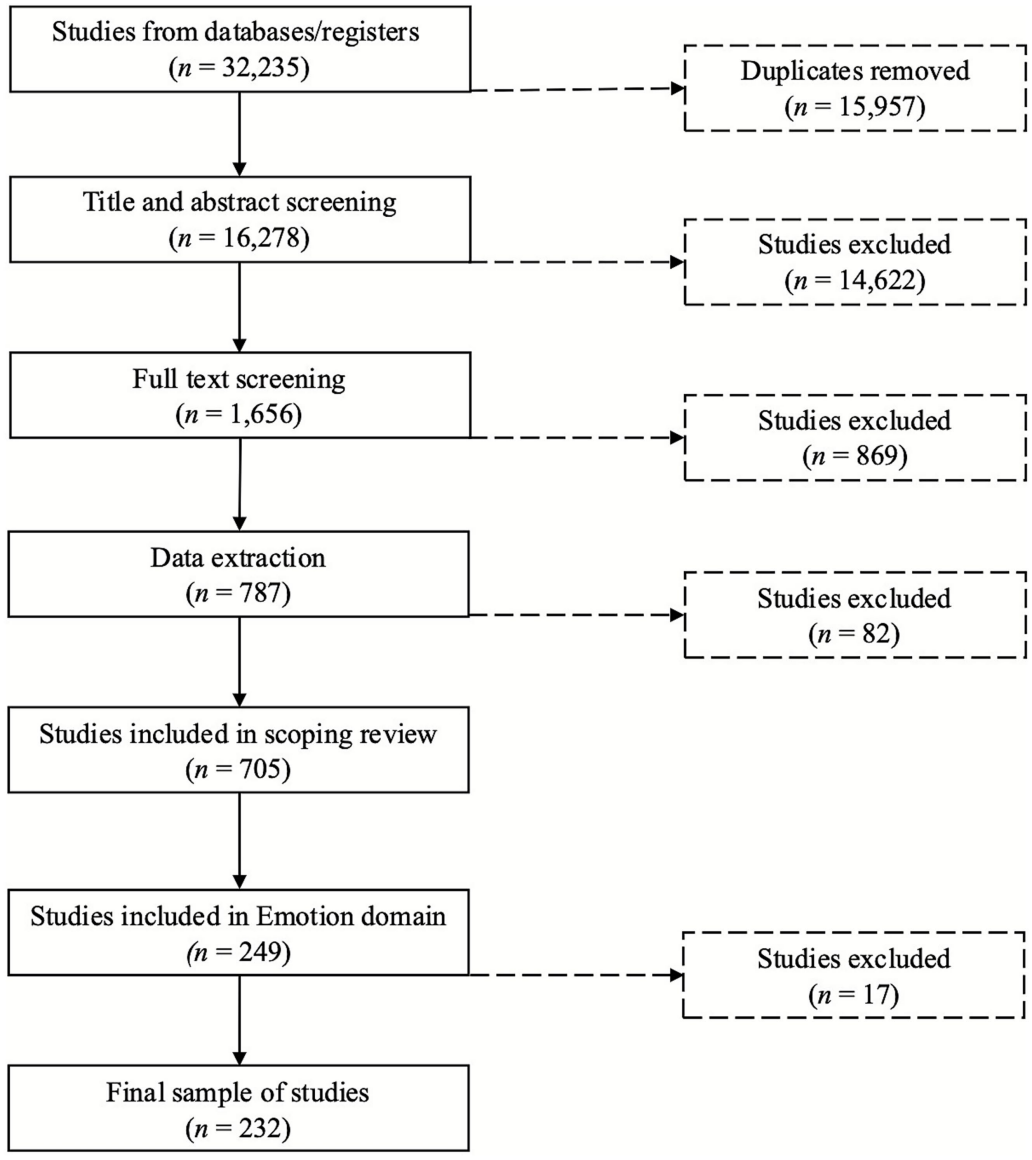
PRISMA flowchart of the article selection process.

A subset of 20 titles was selected for initial training and screening by all reviewers. To participate in the review and extraction process, reviewers were required to achieve a minimum of 85% inter-rater reliability. Screening was carried out in two stages: title and abstract review for initially include/exclude decisions then full text screening for final eligibility assessment. During each stage, reviewers filtered articles as include or exclude. Each article was checked independently by two reviewers and disagreements were resolved by a third “expert” reviewer. A PRISMA flow diagram of screened articles and final sample is provided in Figure 1.

### 2.4 Data extraction and synthesis

Data were extracted via Qualtrics, including sample age, domains mentioned, language of measure, duration, technology or materials requirements, mode of administration, reliability and validity information. Inter-rater agreement was assessed on 31 randomly selected articles, and the agreement ranged from 75.8 to 90.3% (mean Agreement = 81.9%) for sample characteristics (size, age, language, clinical status).

In addition to the quantitative extraction elements, we identified key themes to describe each measure’s purpose. These themes were developed by the first and second author through a process of iterative narrative review similar to what is described in Mays et al. (50). The first author extracted repeated phrases from measure descriptions, generating initial codes. Codes were consolidated into themes when they were identified in at least two unique articles using the same measure. After determining all potential codes and associated themes, the first and second author discussed the findings, adjusting themes to reflect any remaining repeated phrasing that did not neatly fit into the existing themes. Upon discussion and agreement from both authors, the first author re-coded the remaining dataset to associate each measure with one of the eight themes (see Supplementary Table S1 for which themes were applied to each extracted measure).

## 3 Results

### 3.1 Search results

Our initial search yielded 32,235 articles. Once we removed 15,957 duplicate articles, 16,278 articles went through title and abstract screening. After this screening, we were left with 1,656 articles to move on to full text review. Full text review eliminated 869 articles, and we extracted data from 787 articles. During extraction, we removed 82 studies for a final sample of 705. Of these articles, 249 included at least one measure of ENC DM. We then excluded 17 articles in this sample because they used samples of only adults under 45 years old (*n* = 8), did not include age (*n* = 1), or were deemed outside the scope of ENC DM (*n* = 8). Therefore, our final sample includes 232 articles. Among the 232 articles reviewed, 183 (78.9%) examined multiple domains of decision-making (including ENC DM) alongside other areas (functional outcomes, end-of-life, healthcare, financial, and general decision-making abilities). The remaining 49 articles (21.1%) focused exclusively on ENC DM. These articles reported the use of 143 unique decision-making measures. In the results that follow, we provide the original citation for specific measures in-text only the first time they are mentioned.

### 3.2 Sample characteristics

Across 232 articles, sample sizes ranged from 11 to 8,605 participants (median = 86; IQR = 55–201), with over half of the studies (*n* = 131, 56.5%) reporting fewer than 100 participants. All extracted records noted the language in which assessments were administered. If a language was not specified in an article, extractors assumed it was done in the official language of the country where the research took place. Most of the measures were administered exclusively in English (*n* = 102, 43.0%). Measures were also administered in Spanish (*n* = 16, 6.9%), English and another language (*n* = 13, 5.6%), or another, non-English language (*n* = 101, 43.5%). Of the other languages, the most common were German (*n* = 21), Italian (*n* = 10), and Japanese (*n* = 10); all remaining languages appeared in less than 10 articles.

In the reviewed publications, a large proportion of records focused on early to middle adulthood (18 to 64 years of age, *n* = 111, 47.8%) while others covered the adult lifespan (18–85 + years of age, *n* = 77, 33.2%). The remaining publications focused on adults aged 45 and older (16.9%; *n* = 39) or included participants younger than 18 years in addition to adult participants (2.1%; *n* = 5). This review focused on adults aged 45 and older and excluded studies with participants only younger than this age. Collapsing across age categories meant that only 16.9% (*n* = 39) of the articles included in the review had samples consisting exclusively of individuals aged 45 and older, while the remaining 83.1% (*n* = 193) of the articles included participants both below and within the target range.

Nearly half of the studies (*n* = 109, 47.0%) examined both a clinical group and a healthy control group. The remaining studies were almost evenly divided between those that focused exclusively on participants from a special population or with a clinical condition (*n* = 63, 25.9%) and those that included only healthy participants (*n* = 60, 25.9%). The clinical samples were diverse (see Supplementary Table S2 for a full description of groupings), with a predominant focus on psychological disorders (i.e., depression, anxiety, etc.), which accounted for 28.5% (*n* = 49) of the total clinical samples (*n* = 174). Substance use disorder and related usage, including smokers, make up 15.7% (*n* = 27) of the clinical samples mentioned. Neurodegenerative diseases (i.e., MCI, Alzheimer’s, Parkinson’s, Huntington’s, etc.) represented 14.5% (*n* = 25), reflecting a significant area of research interest. Non-clinical special groups constituted 7.6% (*n* = 13), while gambling disorders were present in 7.0% (*n* = 12) of the studies. Neurologic disorders are represented by 6.4% (*n* = 11) of articles, and other clinical conditions, such as cancer, chronic illnesses, autoimmune diseases, and obesity/metabolic syndromes, each made up 2.9% (*n* = 5). Developmental and rare diseases were less common, each comprising 1.7% of the article sample (*n* = 3). Eating disorders as a group were present in 1.2% (*n* = 2) of studies, and there were very few instances where samples overlap conditions or used multiple clinical groups, such as obesity and eating disorders or psychological disorders and substance use. When they did occur was less than 1% (*n* = 1). Supplementary Table S2 in the supplement provides a detailed breakdown of the specific clinical groups categorized under each broad clinical description.

#### 3.2.1. DM and neurodegeneration

Given that the larger ARMCADA project focuses specifically on decision-making related to normative and abnormal cognitive decline, we further analyzed the sample characteristics of papers that included clinical samples with neurodegenerative diseases. Across these 25 articles, sample sizes ranged from 14 to 208 participants (median = 65; IQR = 40–98). Of the total samples, 4.0% (*n* = 1) included participants from the age groups 18–64. A larger portion, 32.0% (*n* = 8), covered a broader range, including participants ranging from 18–84. Additionally, 36.0% (*n* = 9) of the samples comprised individuals from the age groups 45–84. Lastly, another 28.0% (*n* = 7) included participants from 45 to 85 and older. Given the role of aging in cognitive decline, this distribution aligns with our expectations of older age groups in these research samples.

### 3.4 Measure characteristics

**Figure 2:**
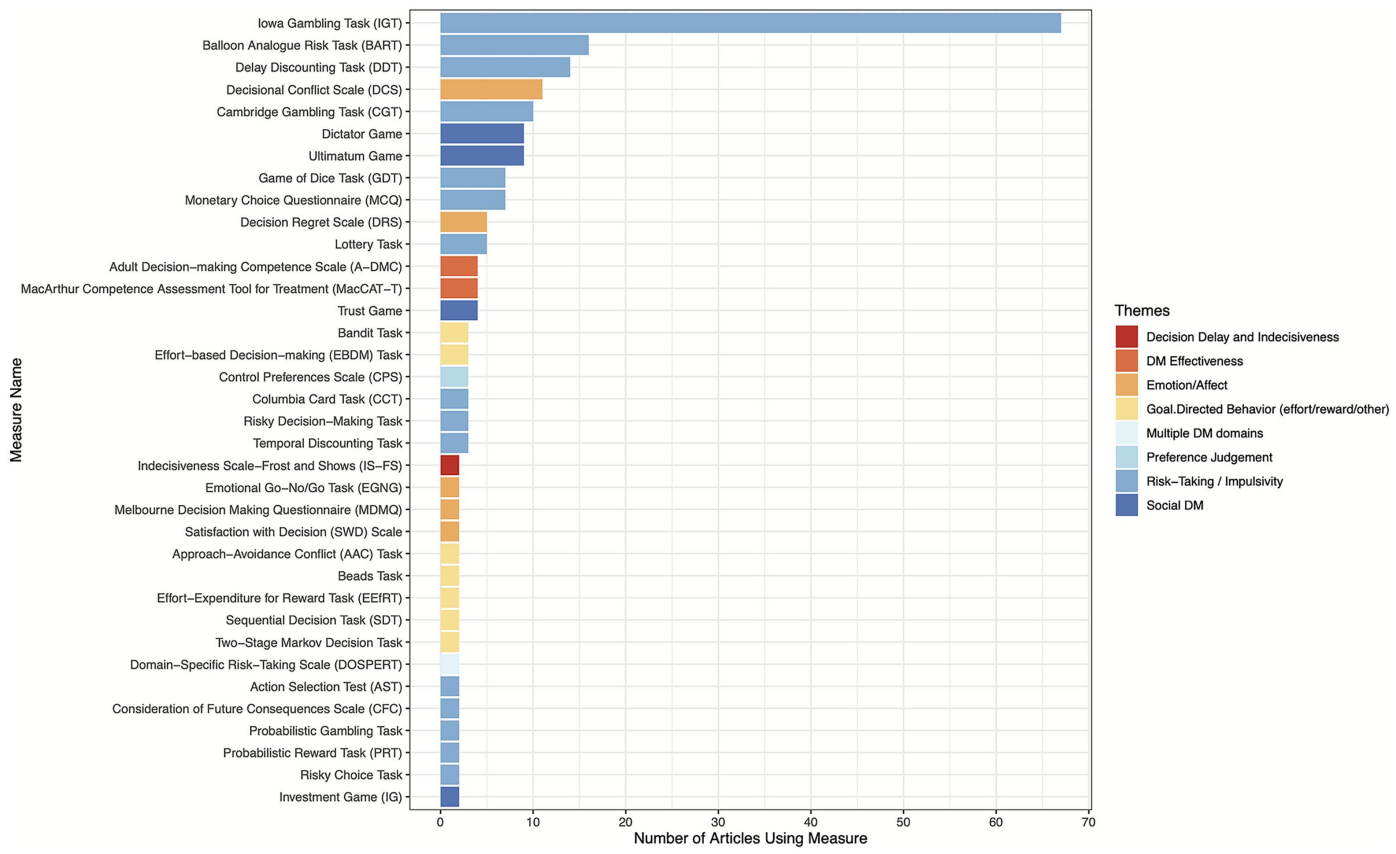
Frequency of mentions for the 36 measures appearing in more than one article, with bar colors denoting the thematic domain associated with each measure. This figure only presents the 36 measures and their corresponding themes which appeared in more than one article. The full list of themes and corresponding measures can be found in Supplementary Table S1.

While a total of 342 measures were identified across the 232 articles, only 143 of these measures were unique. Of the 143 unique measures extracted, the most common measures used in this sample were: Iowa Gambling Task (IGT; (51); *n* = 67 articles), Balloon Analogue Risk Task [BART; (52); *n* = 16 articles], Delay Discounting Task [DDT; (53); *n* = 14 articles], Decisional Conflict Scale [DCS; (54); *n* = 11 articles], and Cambridge Gambling Task [CGT; (55); *n* = 10 articles]. The remaining 139 measures appeared in less than 10 articles each with 74.80% (*n* = 107) of all measures only appearing in a single article. Refer to Figure 2 for a summary of the frequency with which measures appear across multiple articles. Most of the measures were administered in-person (*n* = 235; 68.7%), though some were done exclusively remotely (*n* = 79; 23.1%) where participants did not need to be in the same physical space as the administrator. In general, most of these measures were lab-based digital tasks using computer, smartphone, or iPad (all digital *n* = 252, 73.4%). Semi-structured interviews comprised a smaller portion of this sample at 7.6% (*n* = 26) and paper and pencil measures were an even smaller portion of this sample (*n* = 13; 3.8%).

The results across the 342 measures extracted from each article regarding the reliability and validity of measures indicated a significant lack of information provided. For internal consistency, most measures within articles, 91.2% (*n* = 312), did not include any relevant data. Among the remaining measures included within the articles, 4.7% (*n* = 16) reported internal consistency for measures with subscales and 3.8% (*n* = 13) provided information for samples without subscales. For inter-rater reliability, 100% of measures within articles (*n* = 342) did not provide information. In terms of test–retest reliability, 99.1% of measures within articles (*n* = 339) did not provide information, 0.6% of articles (*n* = 2) reported test–retest reliability for samples without subscales, and 0.3% of article (*n* = 1) provided data for each subscale of the decision-making measure. Regarding validity, a substantial majority, 89.2% of the measures included in articles (*n* = 305), indicated that validity information was not available, while 10.8% (*n* = 37) reported that validity information was provided. Lastly, although information on cross-cultural validation was not widely available, the nine most commonly used measures and all the languages they were administered in, excluding English, is provided in Supplementary Table S3.

Because the studies included in our review reported very few psychometric properties, we conducted a secondary search to locate the original validation sources for all measures used by at least five papers. This resulted in nine unique measures. Findings from this supplemental search are summarized in Table 3.

**Table 3:** Most frequently used measures of emotional and non-cognitive factors in decision-making with associated psychometric properties.

Measure	Frequency (%)	Internal consistency	Test–retest reliability (correlations)
Iowa Gambling Task [IGT; (51)]	67 (20.36%)	0.22–0.55 (78, 79)	0.06–0.37 (80, 81)
Balloon Analogue Risk Task [BART; (52)]	16 (4.86%)	0.70–0.78 (82)	0.50–0.90 (80, 83–85)
Delay Discounting Task [DDT; (53)]	14 (4.26%)	0.89 (86)	0.54–0.57 (83)
Decisional Conflict Scale [DCS; (54)]	11 (3.34%)	0.78–0.92 for total scale, 0.58–0.92 for subscales (54)	0.81 (54)
Cambridge Gambling Task [CGT; (55)]	10 (3.04%)	Unavailable	0.66–0.90 (87)
Dictator Game (88)	9 (2.74%)	Internal consistency cannot be assessed due to being a one-shot game with a single item.	0.63 (89)
Ultimatum Game (90)	9 (2.74%)	Internal consistency cannot be assessed due to being a one-shot game with a single item.	0.60 (89)
Game of Dice Task [GDT; (91)]	7 (2.13%)	Unavailable	0.49 (80)
Monetary Choice Questionnaire [MCQ; (92, 93)]	7 (2.13%)	0.94–0.96 (94)	0.33–0.86 (95–97)

The percentages reported in the second column are based on the 232 articles included in the scoping review and represent the most frequently used measures (*n* > 5) among the 143 unique measures identified. The remaining 134 measures were each used in one to five articles. The psychometric properties listed in the table are taken from original source publications when available and supplemented with subsequent studies as necessary; they were not extracted as part of this scoping review.

Turning back to the primary scoring review results, in a comprehensive analysis of the 143 different decision-making measures, several key themes emerged, highlighting both emotion and non-cognitive dimensions of decision-making (see Figure 2, Supplementary Table S1 for the full list of themes and corresponding measures). The predominant theme, encompassing 32.2% of the measures (*n* = 46), was *Risk-Taking/Impulsivity*, which included tasks assessing tendencies toward engaging in risky behaviors and making hasty decisions without thorough consideration. Notable examples of tasks in this category are the Iowa Gambling Task (IGT), Balloon Analogue Risk Task (BART), and Delay Discounting Task (DDT). Following this category, *Goal-Directed Behavior (effort/reward/other)* represented 17.5% of the measures (*n* = 25), focusing on how individuals weigh effort, engage in reward learning, and pursue goals effectively. Examples of tasks under this theme include the Effort-based Decision-Making Task [EBDM; (56)], Reward Learning Task (57), and Relapse Analogue Task [RAT; (58)]. Measures capturing *Multiple Decision-Making Domains* accounted for 10.5% (*n* = 15), integrating various aspects of decision-making across different contexts, such as the combination of affective and social decision-making or perceptual and value-based decision-making. *Emotion/Affect* comprised 9.8% of the measures (*n* = 14), examining the influence of emotional states and affective responses on decision-making, with tasks such as the Berlin Emotional Responses to Risk Instrument [BERRI; (59)] and the Decisional Conflict Scale (DCS). *Social Decision-Making*, representing 9.1% (*n* = 13), involved tasks assessing decisions within social contexts, including interactions, relationships, trust, and altruism, with examples like the Dictator Game and Minnesota Trust Game [MTG; (60)]. *Perceptual Decision-Making* made up 7.0% (*n* = 10), focusing on decisions based on sensory input and perceptual judgments, including the Coin Task (61) and Line Length Judgment Task (62). *Decision-Making Effectiveness* accounted for 5.6% (*n* = 8), capturing aspects of decision-making competence, confidence, and quality, with examples such as the Adult Decision-Making Competence Scale [A-DMC; (36)] and Decision-Making Quality Index (63). Measures related to *Information Processing, Jumping to Conclusions, and Cognitive Flexibility* represented 4.9% (*n* = 7), assessing how individuals process information and adjust their thinking, with tasks like the Box Task (64) and Postdecision Evidence Integration Task (65). *Decision Delay and Indecisiveness* made up 2.10% (*n* = 3), focusing on the impact of procrastination and uncertainty on decision-making, with measures including the Indecisiveness Scale-Frost and Shows [IS-FS; (66)] and the Indecision Task (67). Finally, *Preference Judgment* accounted for 1.4% (*n* = 2), representing individual differences in preferences, with tasks such as the Preference Judgment Task (68) and the Control Preferences Scale [CPS; (69)]. Collectively, these themes reflect a broad spectrum of factors influencing decision-making, emphasizing both affective and non-cognitive elements.

#### 3.4.1 DM and neurodegeneration

In total, 29 unique measures were used in the 26 articles which included clinical samples with neurodegenerative diseases. The most frequently used decision-making measures were the IGT (10.8%; *n* = 4) and the MacArthur Competence Assessment Tool for Treatment [MacCAT-T; (70); 8.1%; *n* = 3]. Other measures included the DDT, Dictator Game, and Effort-based Decision-making (EBDM) Task, each utilized in 5.4% (*n* = 2) of the studies. The remaining 24 measures were each used by only one article. When examining articles that exclusively involved clinical samples with neurodegenerative diseases, only 7 of the overall 10 themes emerged. The most prevalent theme was *Risk-Taking/Impulsivity*, comprising 34.5% (*n* = 10) of the measures. Following this, *Goal-Directed Behavior (effort/reward/other)* accounted for 24.1% (*n* = 7), and *Social Decision-Making* represented 17.2% (*n* = 5). *Multiple Decision-Making Domains* made up 10.3% (*n* = 3), while *Emotion/Affect* comprised 6.9% (*n* = 2). *Decision-Making Effectiveness* and *Perceptual Decision-Making* each represented 3.5% (*n* = 1) of the measures. These themes, previously observed in the full sample of articles, highlight the diverse range of factors that researchers have focused on over the last 5 years in studies involving individuals with neurodegenerative diseases.

## 4 Discussion

From 32,235 candidate articles, 787 were analyzed, with 232 studies meeting final inclusion criteria, specifically exploring the role of emotion and various non-cognitive factors in decision-making. These recent studies provided valuable insights into the complex interplay between emotional and non-cognitive factors in shaping choices.

The findings of the present review contribute to and expand upon the previous literature by offering an integrative perspective on the diverse dimensions of Emotional and Non-Cognitive influences on Decision-Making (ENC DM). Within decision-making, there was emphasis on risk-taking, impulsivity, and goal-directed behaviors like temporal discounting, which are crucial for understanding how individuals, especially in aging populations, evaluate and respond to potential rewards and risks. In particular, the current analysis identifies Risk-Taking/Impulsivity as the most commonly assessed construct, accounting for over 32% of the measures. This aligns closely with previous reviews (71, 72), which also highlighted the prominence of tasks such as the Iowa Gambling Task (IGT), Ballon Analogue Risk Task (BART), Cambridge Gambling Task (CGT), and Delay Discounting Task (DDT) in research on impulsivity and risk, particularly within emotionally charged contexts and aging.

While risk-taking, impulsivity, and temporal discounting alone provide a solid foundation for assessing decision-making tendencies and individual differences within decision-making, findings from this scoping review reveal that also incorporating emotion and non-cognitive factors, such as affective processing and social decision-making, can add greater context. Affective processing, for example, shapes how emotions influence risk perception and judgment, while social decision-making covers the importance of interpersonal relationships and social contexts in guiding choices (3). Perceptual factors further contribute by affecting how sensory inputs are interpreted, which in turn influences judgment and decision strategies. Risk-taking and impulsivity are also directly tied to decision-making under uncertainty and can be strongly shaped by emotional states, personality traits, and other individual differences such as tolerance to ambiguity (1, 2, 16). Temporal discounting, which reflects the tendency to prefer immediate rewards over delayed ones, is especially significant as it provides insights into how individuals weigh short-term versus long-term outcomes, a process that may be altered in the context of cognitive impairments (8, 9).

This review also emphasizes the complexity of decision-making and the necessity of considering both emotional and non-cognitive dimensions when assessing decision-making capacity, particularly in clinical and non-clinical groups. Thus, understanding emotional and non-cognitive processing is particularly critical because abnormalities in mood and emotional regulation can serve as early neuropsychiatric symptoms (23–25), which are often present in dementia and related neurodegenerative conditions. In aging populations, the interplay between emotions, cognitive control, and reward sensitivity becomes especially important, as emotional states can have a profound impact on decision-making, particularly under conditions of cognitive decline. These findings align with theoretical models such as the Affect-Integration-Motivation framework (22), which proposes that decision behavior in aging reflects changes in affective brain circuitry that influence how individuals weigh risk and reward. Recognizing these affective changes can offer valuable insights into the onset and progression of cognitive impairments.

Elliott et al. (71) further noted that the association between emotion-related impulsivity and risky decision-making is often moderated by arousal, emphasizing that emotional and physiological states play a significant role in shaping decision behavior. Similarly, Rosi et al. (72) emphasized the frequent use of these tasks, alongside emotionally framed scenarios and moral dilemmas, to capture age-related changes in reward sensitivity and affective processing. This consistent use of such tasks in recent years reinforces the importance of individual differences in impulsivity and risk behaviors for understanding how emotion and non-cognitive factors shape decision-making across the lifespan and diverse populations. These patterns resonate with the Emotion-Imbued Choice model (1), which emphasizes that emotions are embedded in decision-making by guiding attention, shaping risk perception, and influencing evaluation of outcomes.

The present study also expands previous research by considering a wider range of decision-making constructs beyond risk and impulsivity. While earlier reviews, such as those by Bartholomeyczik et al. (7) and Perach et al. (73), emphasized the role of emotion in shaping decision behavior, they focused more narrowly on specific mechanisms like emotion regulation or the influence of incidental affect. For example, Bartholomeyczik et al. (7) demonstrated that incidental emotions can significantly alter risk perception and choice behavior, with outcomes influenced by task context, individual emotional reactivity, and cognitive control. These findings resonate with the current study’s identification of Emotion/Affect as a distinct but less frequently assessed theme in recent years (9.8% overall; 6.9% in neurodegenerative samples), suggesting a potential decline in the use of emotion-focused decision-making measures in recent years, despite their continued relevance in both clinical and non-clinical research. While fewer studies focused on decision-making effectiveness and preference judgment, these areas remain critical for achieving a comprehensive understanding of the decision-making process.

Similarly, Mather (74) highlighted how aging and related cognitive changes impact emotional decision-making, particularly through improved emotion regulation and a tendency to prioritize positive outcomes. This positivity bias may help explain why older adults often show reduced sensitivity to negative consequences and changes in risk-taking behavior—patterns also observed in individuals with neurodegenerative diseases. In the current review, studies involving clinical samples with neurodegenerative conditions most frequently assessed Risk-Taking/Impulsivity (34.5%), with tasks such as the IGT and DDT being commonly used. These findings align with previous research on emotional salience and impulsivity in aging and clinical populations (72, 74). Additionally, tasks such as the MacArthur Competence Assessment Tool for Treatment (MacCAT-T) and the Everyday Decision-Making (EBDM) task suggest an interest in how decision-making competence and motivation are impacted in these populations.

Another notable finding was that most measures were administered in person, with a smaller proportion conducted remotely. The majority of assessments involved lab-based digital tasks using computers, smartphones, or iPads, while more traditional approaches, such as semi-structured interviews and paper-and-pencil measures, were used much less frequently. Given this reliance on highly controlled environments, future research should prioritize the development and validation of ecologically valid decision-making tasks that better reflect the dynamic, multifaceted nature of real-world decisions. These tasks should incorporate contextual factors such as time pressure, social influences, and environmental cues. Importantly, to support their use in longitudinal studies aimed at predicting the onset of cognitive impairment, such measures must also be designed for ease of integration and low participant burden while also demonstrating strong psychometric properties (which few of these articles mentioned). This would allow for scalable, repeated administration across time and diverse settings, ultimately improving the sensitivity and utility of decision-making assessments in aging populations.

Taken together, these results emphasize the need to integrate emotional and non-cognitive factors into decision-making frameworks, particularly for populations vulnerable to cognitive decline. These results also coincide with the idea of “ecological fit” (21), which posits that aging individuals shift their decision-making strategies to emphasize emotional wellbeing and practical relevance, rather than cognitive precision, which posits that aging individuals shift their decision-making strategies to emphasize emotional wellbeing and practical relevance, rather than cognitive precision.

Additionally, these results highlight the importance of incorporating decision-making capacity alongside emotion and other non-cognitive factors into neuropsychiatric assessments and dementia care. In clinical practice, assessments of decision-making in older adults often focus primarily on cognitive decline, overlooking meaningful behavioral components such as mood symptoms or personality traits. Because current approaches show substantial heterogeneity in how these components are measured, greater standardization is needed to support consistent clinical use and interpretation. Systematically integrating these ENC factors can provide a more holistic understanding of decision-making, especially in patients with psychiatric or other comorbidities that influence everyday choices. Establishing uniform methods of how clinicians evaluate decision-making capacity, as well as clarifying how ENC factors shape decision-making, would enrich the patients’ clinical profiles and allow for more tailored, effective interventions. Moreover, standardized ENC DM measures also hold important longitudinal value by enabling the identification of predictive markers for individuals at risk of developing ADRD and providing reliable tools for tracking intervention efficacy.

Overall, the scoping review draws attention to the importance of integrating various emotional and non-cognitive constructs to form a comprehensive understanding of decision-making. The studies reviewed indicate that decision-making research across the lifespan often incorporates models that extend beyond purely cognitive frameworks and highlights the relevance of emotional and non-cognitive influences on decision-making. These influences, shaped by both individual differences and age-related changes, are important for understanding decision-making processes. This review examines trends in recent literature in conjunction with theoretical frameworks and related reviews to emphasize the need for a more integrative, adult lifespan-informed approach to understanding the influence of emotional and non-cognitive factors on real-world decision-making. Such integration is critical for advancing assessment tools, tailoring interventions, and enhancing decision support strategies, particularly for populations facing cognitive decline. Although ENC DM was a meaningful area of focus, this review revealed it was most frequently examined simultaneously with other decision-making domains. This pattern points to a broader recognition in the literature: decision-making is multifaceted and deeply influenced by contextual and cognitive-emotional interactions. This underscores the importance of studying ENC DM within a broader, multidimensional framework to fully capture the complexity of real-world decision-making in aging populations.

### 4.1 Limitations

Scoping reviews have inherent limitations, including their reliance on a narrative approach (rather than quantitative) synthesis of data as in systematic reviews or meta-analyses. While this approach allows for broad exploration, future work could benefit from a systematic review or meta-analysis to draw stronger, data-driven conclusions about ENC DM measures. Although a broad set of search terms, developed collaboratively by a large team of scientists to capture decision-making ability was used, the search strategy and final list of terms may still reflect selection bias based on the articles included in this review. Specifically, the inclusion of search terms related to ENC DM may have led to an overrepresentation of certain factors (such as emotional, social, and perceptual ones), which should be considered when interpreting the results. Similarly, the exclusion of certain search terms may have unintentionally led to the omission of factors that are relevant to the field’s focus, such as the cultural and linguistic heterogeneity of the included samples. For example, we acknowledge that the studies part of this scoping review primarily rely on Western sociocultural norms, which can shape what is considered optimal decision-making. Risk tolerance, impulsivity, and reward sensitivity are factors that may vary systematically across different cultures (75, 76). Additionally, ENC and DM may rely on nuanced instructions and hypothetical task scenarios as well as emotionally valenced stimuli, which can result in heavy linguistic and cognitive load, particularly for non-native English speakers. Although similar numbers of studies in our review used English and translated versions of the measures, language of administration does not fully capture cultural context. Moreover, the frequency with which any given language was used becomes even more fragmented when considering the large number of distinct measures identified, further limiting cross-study comparability. Differences in translation quality, cultural norms surrounding decision-making, and culturally patterned response styles may influence how participants interpret items, potentially affecting the validity of measures originally developed in English. Future research should assess measurement invariance across language and cultural groups and consider culturally adapted versions to strengthen validity across diverse contexts. Additionally, by excluding the gray literature, our results may be subject to publication bias and potentially missing key measures that assess ENC decision-making abilities.

Moreover, the review’s exclusion of studies involving individuals younger than 45 years old may have overlooked relevant research on emotion and decision-making in younger populations, which could be pertinent to understanding decision-making across different age groups and the lifespan. Therefore, the emphasis on older adults may not capture the full spectrum of decision-making research in this domain. In addition to future work considering a larger age span for ENC DM measures, future research should focus on measures used in longitudinal designs that apply decision-making measures to individuals at risk for dementia to evaluate their predictive validity and potential utility in early detection. Another limitation of this study was the lack of information on the reliability and validity of the measures used, as well as whether any of them had undergone cross-cultural validation. This gap stemmed largely from insufficient reporting of basic psychometric details across the reviewed articles, particularly of experimental or non-survey based tasks. Although some indices may not be feasible or relevant for all designs, internal consistency of experimental or non-survey tasks can be calculated from a single administration when a measure includes multiple items assessing the same construct (77). Not reporting this fundamental metric limits the interpretability and comparability of findings and highlights the need for more consistent psychometric reporting. To address these issues, future research should systematically assess and report the psychometric properties of the DM measures reviewed by our study by expanding their methods and analysis to incorporate evidence from the measure’s original validation studies. Finally, the review found a broad range of decision-making measures with limited standardization, complicating comparisons and the identification of consistent patterns, something other researchers should recognize when interpreting our findings.

### 4.2 Conclusions and recommendations

This review synthesized 232 articles on ENC DM, revealing a rich and varied landscape of research themes and methodologies. Risk-Taking/Impulsivity emerged as the dominant theme and underscores the importance of understanding how individuals engage in risky behaviors and make impulsive decisions, a key area of interest due to its implications for both normative and impaired cognitive functioning. Goal-Directed Behavior and Multiple Decision-Making Domains also featured prominently, reflecting ongoing efforts to examine how individuals evaluate effort, rewards, and integrate various decision-making factors. Emotion and affect, while well-represented, may have been disproportionately influenced by the selection of search terms, a potential bias by giving more weight to certain constructs. These proportions provide useful insights, they should be interpreted carefully, considering the targeted nature of the search terms used in this review. While most studies included participants across a broad age range and encompassed both clinical and non-clinical groups, the specific themes and measures used emphasize a focused interest in understanding the nuanced impacts of emotion, social context, and other non-cognitive factors on decision-making. Overall, these findings illustrate the complexity of assessing decision-making and the diverse range of research conducted over the past 5 years.

## Data Availability

The original contributions presented in the study are included in the article/Supplementary material, further inquiries can be directed to the corresponding author.
