# Supplementary material for: A scoping review of emotion and non-cognitive measures of decision-making ability in older adults by the ARMCADA study
Source: Front Public Health. 2026 Feb 4;14:1718861. doi: 10.3389/fpubh.2026.1718861 (PMC12913178; doi:10.3389/fpubh.2026.1718861)
Supplement: Supplementary file 1 [file Table_1.docx]

**Table S1. Developed Themes Based on Extracted Measure Descriptions**

| **Measure Themes** | **Measure Name** |
| --- | --- |
| DM Effectiveness | Adult Decision-making Competence Scale (A-DMC) |
|  | Combined Scale for Proxy Informed Consent Decisions (CONCORD scale) |
|  | Competence in Decision Rules (CDR) |
|  | Confidence Task |
|  | Decision Self-Efficacy Scale (DSE) |
|  | Decision-making Quality Index |
|  | MacArthur Competence Assessment Tool for Treatment (MacCAT-T) |
|  | Prediction Question |
| Decision Delay and Indecisiveness | Indecision Task |
|  | Indecisiveness Scale- Germeijs and De Boeck (IS-GDB) |
|  | Indecisiveness Scale-Frost and Shows (IS-FS) |
| Emotion/Affect | Berlin Emotional Responses to Risk Instrument (BERRI) |
|  | Counterfactual Thinking Gambling Task (CGT) |
|  | Decision Regret Scale (DRS) |
|  | Decisional Conflict Scale (DCS) |
|  | Emotional Arousal and Decision Strategies Questionnaire |
|  | Emotional Experience Task |
|  | Emotional Go-No/Go Task (EGNG) |
|  | Emotional Memory Task |
|  | Interpersonal Reactivity Index (IRI) |
|  | Lexical Decision Task (LDT) |
|  | Medical Decision-making Regret (1-item) |
|  | Melbourne Decision Making Questionnaire (MDMQ) |
|  | Satisfaction with Decision (SWD) Scale |
|  | Self-Care Decisions Inventory |
| Goal‐Directed Behavior (effort/reward/other) | Active Information Sampling Task |
|  | Altruistic Intertemporal Choice Task |
|  | Apple Gathering Task |
|  | Approach-Avoidance Conflict (AAC) Task |
|  | Bandit Task |
|  | Beads Task |
|  | Cognitive Effort Discounting (COGED) Task |
|  | Effort Discounting Questionnaire (EDQ) |
|  | Effort Expenditure for Reward Task (EEfRT) |
|  | Effort-Discounting Task (EDT) |
|  | Effort-Expenditure for Reward Task (EEfRT) |
|  | Effort-based Decision-making (EBDM) Task |
|  | Intertemporal Choice Task |
|  | Matching Pennies Task |
|  | Monetary Incentive Delay (MID) Task |
|  | NASA Task Load Index (NASA TLX) |
|  | Pay-it-forward effortful decision-making task |
|  | Punishment Learning Task |
|  | Relapse Analogue Task (RAT) |
|  | Reward Learning Task |
|  | Sequential Decision Task (SDT) |
|  | The Effort-Expenditure for Reward Task (EEfRT) |
|  | Three-Stage Markov Decision Task |
|  | Two-Stage Markov Decision Task |
|  | Willingness to Wait (WTW) Task |
| Information Processing, Jumping to Conclusions, and Cognitive Flexibility | Box Task |
|  | Category Switch Task (CST) |
|  | Information Sampling Task (IST) |
|  | Passive Information Sampling Task |
|  | Postdecision Evidence Integration Task |
|  | Reversal Learning Task (RLT) |
|  | Whodunit Task |
| Multiple DM domains | Adults and Older Adults Functional Assessment Inventory |
|  | Cambridge Neuropsychological Test Automated Battery (CANTAB) |
|  | Client Evaluation of Self and Treatment (CEST) |
|  | Decision Making Tendency Inventory (DMTI) |
|  | Decision-making Scale (COGSOC) |
|  | Domain-Specific Risk-Taking Scale (DOSPERT) |
|  | Functioning Assessment Short Test (FAST) |
|  | Huntington Disease Rating Scale (UHDRS) |
|  | Maximization Scale |
|  | Multi-step Decision-making Task |
|  | Perceptual and Value-Based Decision-Making Task (PVDM) |
|  | Risk Perceptions Scale |
|  | Stochastically Rewarded Decision-making Task |
|  | Sunk Cost Task |
|  | Vividness of Decision Outcomes |
| Perceptual DM | Cognitive Bias Task (CBT) |
|  | Coin Task |
|  | Color Judgment Task |
|  | Driving Simulator Task |
|  | Flashing Grid Task |
|  | Likelihood Task |
|  | Line Length Judgment Task |
|  | Object Size Comparison Task |
|  | Random Dot Motion (RDM) Task |
|  | Stokings of Cambridge (SoC) |
| Preference Judgement | Control Preferences Scale (CPS) |
|  | Preference Judgement Task |
| Risk-Taking / Impulsivity | Action Selection Test (AST) |
|  | Adjusting Amount Discounting Task |
|  | Ambiguity Tolerance Task |
|  | Balloon Analogue Risk Task (BART) |
|  | Bangor Gambling Task (BGT) |
|  | Barratt Impulsivity Scale (BIS) |
|  | Cambridge Gambling Task (CGT) |
|  | Cards and Lottery Task (CLT) |
|  | Choice Task |
|  | Columbia Card Task (CCT) |
|  | Consideration of Future Consequences Scale (CFC) |
|  | Decision to Drive (2-pts) |
|  | Decision-Making Questionnaire (DMQ) |
|  | Decisions from Experience Task 1 |
|  | Decisions from Experience Task 2 |
|  | Delay Discounting Task (DDT) |
|  | Die Roll Decision-making Paradigm |
|  | Door Opening Task |
|  | Failure to Minimize Risk Assessment |
|  | Financial Risky Choice Paradigm |
|  | Forced Choice Problems Task |
|  | Gamble Acceptance Task |
|  | Gambling Task |
|  | Gambling Task (Slot Machine) |
|  | Game of Dice Task (GDT) |
|  | Health Context Interaction Experiment |
|  | Incentive-Compatible Risk Preference Task (fMRI) |
|  | Iowa Gambling Task (IGT) |
|  | Lottery Task |
|  | Medication Use Decision Scale |
|  | Mixed-Gambles Task |
|  | Monetary Choice Questionnaire (MCQ) |
|  | Motor Gambling Task |
|  | Probabilistic Gambling Task |
|  | Probabilistic Reward Task (PRT) |
|  | Probability-Associated Gambling Task (PAG) |
|  | Risk Choice Experiment Survey (25-items) |
|  | Risk Discounting Task |
|  | Risk Tolerance Task |
|  | Risk-Based Decision-making Task |
|  | Risky Choice Task |
|  | Risky Decision Scenario Task |
|  | Risky Decision-Making Task |
|  | Temporal Discounting Task |
|  | Willingness to pay to reduce one’s risk (WTP) |
|  | Willingness to take the risk (WTT) |
| Social DM | Altruistic Decision Task |
|  | Computer and Human Mediator Neuroeconomics Experiment (Economic Trust Game) |
|  | Costly Punishment Task |
|  | Dictator Game |
|  | Investment Game (IG) |
|  | Minnesota Trust Game (MTG) |
|  | Prisoner’s Dilemma Game (PDG) |
|  | Pro-social Decision-making Task |
|  | Scam Awareness Task |
|  | Social Cognition Battery (COGSOC) |
|  | Social Decision-making and Self-Estimation Task |
|  | Trust Game |
|  | Ultimatum Game |
