# Supplementary material for: A scoping review of emotion and non-cognitive measures of decision-making ability in older adults by the ARMCADA study
Source: Front Public Health. 2026 Feb 4;14:1718861. doi: 10.3389/fpubh.2026.1718861 (PMC12913178; doi:10.3389/fpubh.2026.1718861)
Supplement: Supplementary file 2 [file Table_2.docx]

**Table S2. Descriptions of Broader Clinical or Special Interest Group Categories**

| **Clinical or Special Interest Group Category** | **Description of Group** |
| --- | --- |
| Cancer | BRCA1/2-positive women |
|  | Japanese females with BC/survivors |
|  | Patients w/ early stage lung cancer |
|  | Patients w/ localized prostate cancer |
|  | cancer survivors |
| Chronic Illness / Autoimmune Diseases | Adults w/ at least 1 chronic condition |
|  | HIV positive participants |
|  | HIV positive subjects (n=38) + HC (n=30) |
|  | Participants that are HIV positive |
|  | diagnosed with fibromyalgia |
| Developmental disorders | ADHD + healthy |
|  | Individuals w/ autism |
|  | adults with ADHD |
| Eating Disorder | Binge Eating Disorder/ Co-morbid Binge Eating Disorder and Compulsive Buying |
|  | outpatients with eating disorders |
| Gamblers / Gambling Disorder | Gambler |
|  | Gambling Disorder |
|  | Males w/ Gambling Disorder |
|  | Non-treatment seeking adults with mixed gambling history |
|  | casino patrons/ potential problem gamblers |
|  | gambling disorder |
|  | patients diagnosed with gambling disorder |
|  | patients with gambling disorder |
|  | people with gambling disorder |
|  | t male participants diagnosed with gambling disorder |
| Neurodegenerative diseases | ALS patients |
|  | Huntington's disease; schizophrenia |
|  | Huntingtons Disease including Manifest Group, carriers near to disease onset, and carriers far to disease onset |
|  | MCI |
|  | One of Five Studies included adults with Parkinson's disease |
|  | Parkinson's Disease |
|  | Parkinson's Disease (PD) |
|  | Parkinson’s disease |
|  | Participants w/ Mild to Moderate Alzheimer's Disease |
|  | Patients w/ Alzheimers and Patients with behavioral-variant frontotemporal dementia |
|  | Patients w/ Huntington's Disease |
|  | Patients w/ Parkinson's Disease (w/ STN-DBS and w/o STN- DBS) |
|  | Patients w/ early onset Parkinson's Disease |
|  | Patients w/ idiopathic Parkinson's Disease |
|  | Patients w/ idiopathic Parkinson's Disease w/DBS treatment |
|  | Patients w/ neurodegenerative diseases (Alzheimer’s, frontotemporal dementia, Huntington's disease, dementia w/ Lewy bodies. |
|  | Patients with Parkinson's Disease and Patients with Parkinson's Disease and Impulse Control Disorders |
|  | People w/ mild to moderate Parkinson's disease |
|  | People with Alzheimer’s (their caregivers were also included for certain measures in which only caregivers reported) |
|  | Persons w/ dementia |
|  | mild cognitive impairment |
|  | participants with Parkinson’s disease |
|  | patients w/ behavioral variant frontotemporal dementia; patients w/ Alzheimer’s disease |
|  | pre-manifest Huntington's disease |
|  | Individuals w/ Orbitofrontal cortex damage |
|  | Patients w/ Multiple Sclerosis |
|  | Patients w/ cerebral small vessel disease |
|  | Patients w/ focal-frontal brain lesions (VMPFC and DLPFC) |
|  | Patients w/ frontal lobe lesions (dorsolateral/ventromedial) |
|  | Patients w/ mesial temporal lobe epilepsy with hippocampal sclerosis and Patients w/ posterior cortex epilepsy |
|  | Patients w/ vmPFC damage |
|  | Patients who have had a cerebellar stroke within 1 month-3.5 years of the study |
|  | mesial temporal lobe epilepsy patients with unilateral hippocampal sclerosis |
|  | people with synesthesia |
|  | relapsing–remitting multiple sclerosis |
| Non-clinical Special Group | Dieters |
|  | First-Time Child Exploitation Material Offenders |
|  | Incarcerated offenders/non-offenders |
|  | Pandemic-related worry |
|  | Sleep deprivation |
|  | Social Media Users |
|  | Surrogate decision makers |
|  | drivers w/ DUI convictions |
|  | ex-offenders recently released from jail or prison |
|  | group administered alcohol |
|  | male intimate partner violence perpetrators |
|  | men who sleep with men and transgender women taking PrEP |
|  | tai chi practitioners |
| Obesity/Diabetes/Other Metabolic Syndromes | Diabetes: With glycemic control/without glycemic control |
|  | Obesity |
|  | Ps grouped according to BMI: lean (BMI <= 25) or overweight/obese (BMI > 25) |
|  | bariatric surgery patients |
|  | obese patients preparing to undergo bariatric surgery |
|  | samples with Binge eating disorder (BED) and obese controls |
| Other Clinical | End-Stage Renal Disease |
|  | Insomnia |
|  | Paranoid thinking |
|  | Participants w/ chronic migraine and participants w/ chronic migraine + medication overuse headaches |
|  | Patients diagnosed with age-related cataract (no surgery) |
|  | People undergoing gender reassignment surgery (transmen) |
|  | People who have experienced chronic pain |
|  | Special sample were participants from a Genetics of Impulsivity project |
|  | diagnosed with insular gliomas and had recovered from insular resection |
|  | subjective cognitive impairment + healthy controls |
| Psychological disorders | Adults w/ OCD |
|  | Bipolar Disorder |
|  | Depressed individuals |
|  | Depression and HC |
|  | Depression/SA |
|  | Diagnosis of bipolar disorder (BD) with recurring cycles of manic symptoms (BD-I) |
|  | Individuals w/ hoarding disorder |
|  | MDD |
|  | MDD/Suicide Attempt |
|  | Major depressive disorder |
|  | OCD + HC |
|  | Offenders w/ antisocial personality disorder |
|  | Participants diagnosed w/ major depressive disorder |
|  | Participants w/ anxiety/ depression |
|  | Participants w/ schizophrenia or schizoaffective disorder (SZ) |
|  | Participants w/ schizophrenia/schizoaffective disorder |
|  | Patients w/ Obsessive Compulsive Disorder (OCD) |
|  | Patients w/ major depressive disorder |
|  | Patients w/ schizophrenia |
|  | Patients w/ schizophrenia, bipolar disorder, and major depressive disorder |
|  | Patients w/ schizophrenia/ schizoaffective disorder |
|  | Patients w/ schizophrenia/schizoaffective disorder |
|  | Patients w/ schizophrenia/schizoaffective disorder (N = 33), bipolar disorder (N = 47), unipolar depression (N=61) |
|  | Patients w/ treatment resistant major depressive disorder |
|  | Patients w/ treatment resistant schizophrenia |
|  | People w/ OCD |
|  | People w/ narcissistic personality disorder |
|  | People w/ schizophrenia/schizoaffective disorders and a separate sample of monozygotic twins |
|  | Persons with schizophrenia or schizoaffective disorder |
|  | SA |
|  | Schizophrenia or Depression |
|  | Schizotypal |
|  | Trauma exposed cases |
|  | Veterans w/ PTSD |
|  | current diagnosis of a major depressive episode (MDE) as part of MDD |
|  | depressed in-patients w/ recent suicide attempt; depressed in-patients w/o history of suicide attempt |
|  | individuals with schizophrenia or schizoaffective disorder |
|  | major depression |
|  | major depressive disorder with and without suicide attempt |
|  | male combat veterans with penetrating traumatic brain injury |
|  | male outpatients with schizophrenia |
|  | patients with schizophrenia |
|  | primary diagnosis of schizophrenia spectrum disorders (those with and without and history of significant interpersonal violence) |
|  | schizophrenia outpatients |
|  | schizophrenia/schizoaffective disorder (SZ), Bipolar 1 Disorder, major depressive disorder, healthy controls |
|  | those with Bipolar Disorder |
|  | People w/ depression and/or anxiety; people w/ substance use disorders |
| Rare diseases | Patients w/ Korsakoff's syndrome |
|  | Patients w/ myotonic dystrophy type-1 |
|  | Patients with CD (who started CBG/CBG naive) |
| Substance Use/SUD/Smokers | AUD |
|  | Chronic cocaine users |
|  | Cigarette Smokers |
|  | Cigarette smokers |
|  | Cocaine users |
|  | Current/former smokers |
|  | Heroin users either undergoing methadone treatment or substance free therapy |
|  | Individuals seeking treatment for cocaine-use disorder |
|  | Individuals w/ opioid dependence and individuals with cannabis dependence |
|  | Opiate Users |
|  | Participants w/ alcohol use disorder |
|  | People w/ alcohol use disorder |
|  | People w/ methamphetamine use disorder |
|  | Persons w/ Cocaine Use Disorder, Cannabis Use Disorder, or Opioid Use Disorder (divided into groups based on substance of choice) |
|  | SUD |
|  | Smokers |
|  | Smokers/ Non-smokers |
|  | co-occurring mental health and substance use disorders |
|  | current DSM-IV diagnosis of alcohol dependence |
|  | current cocaine users |
|  | inpatient alcohol use disorder patients |
|  | inpatient for addiction (cocaine, alcohol, cannabis, heroin) |
|  | justice-involved people receiving treatment for a substance use disorder |
|  | opioid dependence diagnosis |
|  | patients w/ substance dependence disorders or (chronic/heavy) substance abusers |
|  | patients with substance use-related disorders |
|  | present a diagnosis of alcohol or cocaine use disorder |
|  | μ-Opioid Receptor Gene |
