# Supplementary material for: A scoping review of emotion and non-cognitive measures of decision-making ability in older adults by the ARMCADA study
Source: Front Public Health. 2026 Feb 4;14:1718861. doi: 10.3389/fpubh.2026.1718861 (PMC12913178; doi:10.3389/fpubh.2026.1718861)
Supplement: Supplementary file 3 [file Table_3.docx]

**Table S3. Summary of the Frequency Languages (non-English) Were Used for the Top 9 Emotion and Non-Cognitive Decision-Making Measures**

| **Measure** | **Administration Language** | **n** |
| --- | --- | --- |
| Iowa Gambling Task (IGT) | Bulgarian | 1 |
|  | Chinese | 6 |
|  | Farsi | 1 |
|  | French | 6 |
|  | German | 3 |
|  | Greek | 1 |
|  | Hungarian | 2 |
|  | Italian | 8 |
|  | Japanese | 1 |
|  | Portuguese | 3 |
|  | Spanish | 11 |
|  | Taiwanese | 3 |
|  | Turkish | 3 |
| Balloon Analog Risk Task (BART) | Chinese | 1 |
|  | French | 2 |
|  | German | 1 |
|  | Italian | 3 |
|  | Swedish | 1 |
| Delay Discounting Task (DDT) | Chinese | 1 |
|  | Portuguese | 1 |
|  | Spanish | 1 |
| Decisional Conflict Scale (DCS) | Chinese | 2 |
|  | Dutch | 1 |
|  | German | 1 |
|  | Japanese | 1 |
| Cambridge Gambling Task (CGT) | Bulgarian | 1 |
|  | Chinese | 3 |
|  | German | 1 |
|  | Italian | 3 |
|  | Polish | 1 |
|  | Spanish | 1 |
|  | Swedish | 1 |
|  | Taiwanese | 2 |
|  | Turkish | 1 |
| Dictator Game | German | 2 |
|  | Japanese | 1 |
|  | Swedish | 2 |
| Ultimatum Game | German | 2 |
|  | Japanese | 2 |
|  | Swedish | 1 |
| Game of Dice | French | 1 |
|  | German | 1 |
|  | Portuguese | 1 |
| Monetary Choice Questionnaire (MCQ) | Bulgarian | 1 |
|  | Farsi | 1 |

*Note.* These are the languages—other than English—in which the nine most commonly used measures were administered, suggesting possible cultural adaptation. n indicates the number of times the measure was administered in the given language.
